# A whole-body Fast Field-Cycling scanner for clinical molecular imaging studies

**DOI:** 10.1038/s41598-019-46648-0

**Published:** 2019-07-18

**Authors:** Lionel M. Broche, P. James Ross, Gareth R. Davies, Mary-Joan MacLeod, David J. Lurie

**Affiliations:** 1 Aberdeen Biomedical Imaging Centre, University of Aberdeen, Foresterhill, AB25 2ZD Aberdeen UK; 2 Acute Stroke Unit, Aberdeen Royal Infirmary, Foresterhill, AB25 2ZD Aberdeen UK

**Keywords:** Biomarkers, Translational research, Biomedical engineering

## Abstract

Fast Field-Cycling (FFC) is a well-established Nuclear Magnetic Resonance (NMR) technique that exploits varying magnetic fields to quantify molecular motion over a wide range of time scales, providing rich structural information from nanometres to micrometres, non-invasively. Previous work demonstrated great potential for FFC-NMR biomarkers in medical applications; our research group has now ported this technology to medical imaging by designing a whole-body FFC Magnetic Resonance Imaging (FFC-MRI) scanner capable of performing accurate measurements non-invasively over the entire body, using signals from water and fat protons. This is a unique tool to explore new biomarkers related to disease-induced tissue remodelling. Our approach required making radical changes in the design, construction and control of MRI hardware so that the magnetic field is switched within 12.5 ms to reach any field strength from 50 μT to 0.2 T, providing clinically useful images within minutes. Pilot studies demonstrated endogenous field-dependant contrast in biological tissues in good agreement with reference data from other imaging modalities, confirming that our system can perform multiscale structural imaging of biological tissues, from nanometres to micrometres. It is now possible to confirm *ex vivo* results obtained from previous clinical studies, offering applications in diagnosis, staging and monitoring treatment for cancer, stroke, osteoarthritis and oedema.

## Introduction

**Figure 1:**
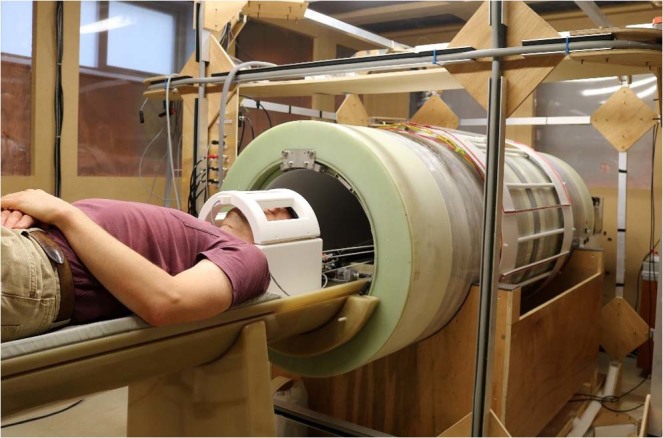
Front view of the FFC-MRI scanner presented here. The scanner has a bore of 50 cm and is surrounded by three pairs of Helmholtz coils for the correction of the Earth’s magnetic field (square section coils visible around the scanner). A volunteer can be seen on the patient table, his head within a birdcage head coil, prior to insertion into the bore of the main magnet.

Early detection and characterisation of disease presents important challenges for clinical research, from degenerative brain disease to osteoarthritis or cancer, despite considerable research efforts over decades, using a variety of technologies. A new kind of diagnostic information is required that can complement the toolbox already available to the clinician to better visualise pathology, improve the understanding of disease mechanisms and to guide and monitor treatment. Here we report on Fast Field-Cycling Magnetic Resonance Imaging (FFC-MRI, see Fig. 1), a new imaging technology for the investigation of soft tissue diseases. It is capable of characterising the molecular dynamics of water and fat over four decades of time-scales, *in vivo* and non-invasively, thereby providing novel, clinically relevant information that complements what is available from existing imaging modalities.

**Figure 2:**
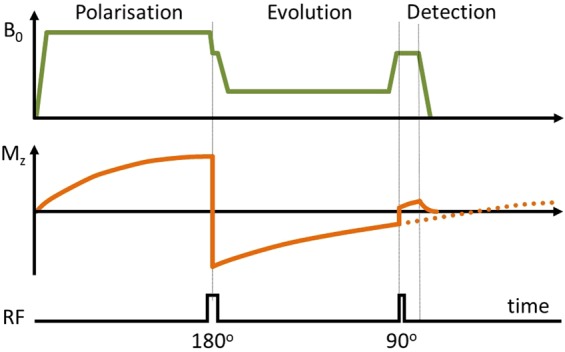
Pulse sequence diagram of a fast field-cycling inversion-recovery pulse sequence. A polarisation period is included to generate magnetisation, which is inverted, after which the spin system is left to evolve at a selected evolution field before the NMR acquisition is made during the detection period using a radiofrequency coil tuned to a frequency appropriate to the detection field strength. Here an inversion pulse is used to improve the dynamics of the NMR signal, note that the Larmor frequency must match the detection coil frequency during the inversion pulse, hence the short plateau at detection strength.

FFC is a technique that has been used in nuclear magnetic resonance (NMR) for many decades^1–11^. FFC-NMR relaxometry is a well-established non-imaging technique that involves varying the strength of the magnetic field applied to a sample, allowing measurement of the longitudinal relaxation time, *T*_1_, as a function of the applied magnetic field strength (Fig. 2). This is in contrast to the stable magnetic field employed in most NMR spectrometers. The result of an FFC experiment is typically reported as a graph of *T*_1_ versus magnetic field, called the NMR dispersion (NMRD) profile. NMRD profiles are extremely informative because *T*_1_ is closely linked to molecular motion: magnetic relaxation is driven by incoherent microscopic motions naturally present in materials due to thermal fluctuations. Protons, which are the most common target for MRI, have a spin of ½ and similarly to other magnetically active nuclei their *T*_1_ relaxation is driven by motions occurring at a specific frequency, the Larmor frequency, which is determined by the strength of the magnetic field via the effect of magnetic resonance. The Larmor frequency ω_L_ is directly proportional to the magnetic field strength B_0_ via the gyromagnetic ratio γ, as follows:

$${\omega }_{L}=\gamma {B}_{0}$$

The gyromagnetic ratio of protons (γ/2π) is 42.578 MHz/T so their NMR Larmor frequency at 1.5 Tesla is 64 MHz and only *ca*. 2 kHz at the Earth’s magnetic field, *ca*. 50 µT. Hence large variations of magnetic field strength give access to a wide range of motional timescales, through *T*_1_ relaxation. Measurement of *T*_1_ on a conventional (fixed-field) NMR or MRI device can only report on a single characteristic time of molecular motion, while FFC allows access to several decades^10^ of motional dynamics, typically from tens of nanoseconds to several milliseconds. Such measurements of the *T*_1_ dispersion with the magnetic field strength, the *T*_1_ NMRD profiles, report quantitatively on the nature of molecular motion and are exploited to determine structural parameters^12–14^. In biological tissues, such time scales correspond to structures varying in size from tens of nanometres up to several micrometres, which is particularly relevant for the study of tissue remodelling in disease.

Other sources of image contrast do exist on fixed-field MRI devices that extend the possibilities of experimentation on water dynamics, mainly *T*_1_-rho, diffusion-weighted MRI (DW-MRI) or quantitative magnetisation transfer^15^, but they offer limited information in this context. *T*_1_-rho is restricted to a rather narrow bandwidth with a maximum frequency limited to *ca*. 2 kHz by excessive non-resonant RF absorption and a minimum frequency of several hundreds of Hz limited by field homogeneity, while DW-MRI on humans is limited for patient safety by gradient field switching (20T/s) and is mostly used *in vivo* to explore phenomena above hundreds of micrometres. FFC-MRI is therefore the only method currently available to explore molecular dynamics non-invasively over such a wide band, offering a hitherto-unavailable window of visualisation into biological structures.

Several authors have previously reported scanners and techniques that exploit FFC contrast, with varying degrees of success, all of them illustrating the technological challenges of integrating FFC into MRI. MRI relaxometry, presented by Carlson *et al*. in 1992^16^, employed a field-offset magnet coil in a low-field clinical scanner and succeeded in generating NMRD profiles of a volunteer’s brain, fat, bone marrow and muscle. However, the magnetic field range was relatively small and, more importantly, the *T*_1_ dispersion measurements obtained were not consistent with theory and results from other studies. In particular, the NMRD profiles of fat and bone marrow showed positive slopes, which do not agree with experimentation on fatty tissues or theory^17^ and, while the NMRD profiles of muscle showed quadrupolar cross-relaxation^18^, the accuracy of the NMRD profile measurement was poor and likely degraded by various stability problems. Another approach was the pre-polarised MRI system developed at Stanford University^19,20^ that exposed the sample to transitory high magnetic field to boost signal strength, followed by measurement of NMR signals at the Earth’s magnetic field or slightly above. Detection at such low fields allowed the use of relatively inexpensive equipment but posed specific challenges related to NMR signal detection at ultra-low frequencies and, even though the results were impressive, research efforts appear to have stalled before reaching human whole-body size. An alternative approach is Delta Relaxation Enhanced MRI (dreMR), which seeks to detect exogenous contrast agents via their *T*_1_-dispersion at high magnetic field, typically ±0.2 T centred on 1.5 T or 3.0 T^21–23^. This approach is very different from ours as it operates at high magnetic fields only, relies on the use of exogenous contrast agents and also requires inserting a field-offset magnet into a pre-existing MRI scanner, which limits the volume of the objects that can be scanned. Data from dreMR are therefore informing on different aspects of tissues than FFC-MRI as performed by our group.

The whole-body human-sized FFC-MRI system that we present here is a breakthrough in MRI technology as it can access magnetic fields from 50 µT to 0.2 T, thereby exploring almost four decades of molecular-dynamics time scales, from 0.5 ms to 100 ns. FFC-MRI provides a rich source of molecular and multi-scale information that is inaccessible by conventional MRI, including *T*_1_-dispersion as well as quadrupolar cross-relaxation^18,24^, that allows quantifying tissue remodelling *in vivo* and non-invasively. Our scanner acquires data at 0.2 T and produces images of sufficient quality to allow clinical assessment with realistic patient scan times. The design that we present here is that of a single, low-inductance, whole-body electromagnet that produces the main magnetic field, driven by fast and powerful current amplifiers. Below we present the system, its performance and limitations, and we propose a perspective on how it can be used for clinical research with examples from a pilot study on acute stroke (Potential Use of Fast Field-cycling IN Stroke, the PUFFINS study). Details of the FFC-MRI system are provided in the Methods section while the Results section describes the performance of the overall device.

## Results

### Field stability and homogeneity

A physical description of the FFC-MRI scanner is presented in the Methods section. The scanner’s field strength (*B*_0_) could be set to any value between 50 μT and 0.2 T, provided that an overall time-averaged duty cycle of 50% or less was adhered to in order to avoid overheating the current-supply amplifiers feeding the main magnet; fixed-field (100% duty cycle) operation was possible at field strengths up to 0.13 T. The system could generate *B*_0_ ramps of 16.5 T/s corresponding to a passage from zero to 0.2 T in 12.1 ms. However, we limited the field ramp to 12 T/s during scans in order to remain comfortably within safety guidelines (IEC 60601-2-33) for the avoidance of peripheral nerve stimulation (typically 20 T/s). Some ringing of the magnetic field was observed after fast *B*_0_ transitions, with a typical decay time of 4 ms, however the effect became negligible for signal acquisition occurring within 15 ms after the end of a ramp. This made a total ramp time and stabilisation delay of less than 30 ms, which is 2- to 20-times shorter than the typical *T*_1_ values at 0.2 T or below for biological tissues.

Temporal field stability at 0.2 T was degraded by random current fluctuations reaching up to 1.5 ppm and spanning over 2 kHz bandwidth, with large peaks at multiples of 50 Hz and particularly at 300 Hz, indicating a likely origin from the mains electrical supplies (300 Hz harmonics are typical of three-phase switched-mode power supplies, operating at 50 Hz frequency in the UK). These produced visible image artefacts (ghosts) that were corrected by a previously-published post-processing algorithm^25^. In addition to fast random fluctuations we also observed slow *B*_0_ drifts due to heating of the main magnet and power electronics, producing monotonously-increasing field variations of up to 500 ppm over 45 minutes. We also detected minor day-to-day variations of the environmental field, typically 50 nT over 24 h, and fast time-varying environmental fields due to the mains electrical cables around the building, typically below 50 nT. The drift due to heating was mainly a problem for the reproducibility of the slice position during image acquisition but the temperature offset correction techniques implemented in the software and presented in the Methods section allowed reaching a precision of 15 ppm for the average temporal stability of the *B*_0_ field, which was enough to avoid the problem entirely. Weekly shimming procedures (see methods) reliably produced field homogeneity of 40 ppm over a cylindrical volume of 30 cm diameter by 15 cm along the magnetic field. It should be noted that FID measurements showed no effect from eddy currents from the variations of *B*_0_, within the limit of the ringing time of the power supplies (10 ms).

### *T*_1_ dispersion-based contrast

**Figure 3:**
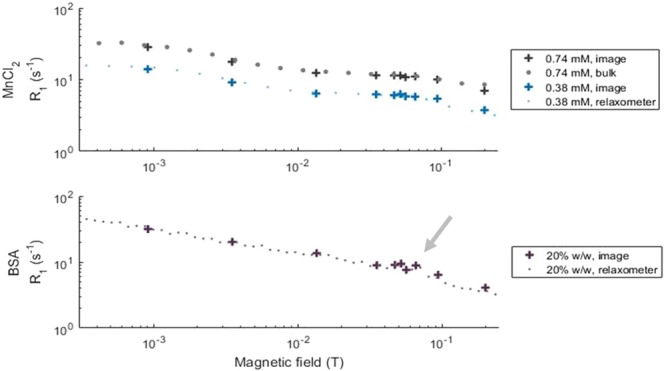
Dispersion profiles of the phantom solutions of manganese chloride (top) and cross-linked BSA (bottom) at 21 °C obtained using the benchtop relaxometer (small dots) and the whole-body scanner (crosses for imaging data, large dots for bulk sample data). The results are in excellent agreement. One can also notice the quadrupolar peaks in the BSA samples around 60 mT (indicated by the grey arrow).

The pulse sequence found to be the most reliable for patient scans to date was a field-cycling spin-echo (FCSE). Calibrations and localisation were routinely performed within the first 5 min of the examination. The bulk measurement of NMRD profiles using test objects, or phantoms (Fig. 3, bulk data, see the Methods section for details) provided results comparable to that of a commercial FFC-NMR relaxometer (Fig. 3, relaxometer data) within 2%, validating the device’s performance for non-imaging protocols. FFC relaxometry data are usually expressed as relaxation rate *R*_1_ = 1/*T*_1_ and *R*_1_ measurements obtained from averaging data over selected regions of images using the same phantom also provided comparable results with an accuracy of 10% (Fig. 3, crosses).

**Figure 4:**
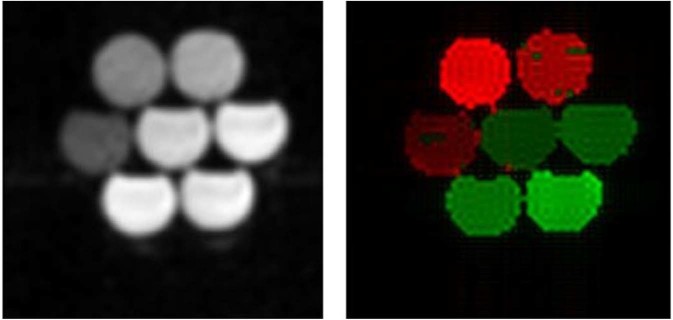
Left: magnitude image of the phantom containing the solutions of MnCl2 and cross-linked BSA, obtained at 0.2 T. Right: concentration image obtained from voxel-wise clustering based on T_1_ dispersion features (dispersion between minimum and maximum field, dispersion at 0.02 T and amplitude of the quadrupolar peaks; red: BSA, green: manganese chloride). The concentration was measured from the dispersion offset compared with a known reference (20% BSA or 0.74 mM MnCl2).

It is well known that a change in concentration of a relaxation agent only affects the vertical offset of the dispersion curve but generally preserves its shape, which can be used to detect the type of material under study. This was performed using automated segmentation, as presented in the Methods section. Figure 4 shows a magnitude image of the bottle phantom acquired at 0.2 T (left) together with a concentration image (right) obtained by analysing the *T*_1_ dispersion curves. This approach successfully differentiated between bottles containing BSA (which mimics biological tissues) and those containing MnCl_2_ (a well-known low-field contrast agent) and provided the correct concentrations within 20%, validating the ability of FFC-MRI to separate compounds based on molecular-dynamics information and to provide quantitative measurements (Table 1). One can note a slight negative bias in the *R*_1_ values measured by FFC-MRI for MnCl_2_ samples.

**Table 1:** Concentration measured from the dispersion image of the bottle phantom.

Sample (units)	BSA (%w/w)			MnCl_2_ (mM)			
Concentration	5	10	20	0.38	0.52	0.74	1.00
Value measured from image	5.3 ± 1.6	11.8 ± 2.8	18.3 ± 0.6	0.346 ± 0.007	0.47 ± 0.01	0.642 ± 0.007	0.94 ± 0.04

### *In vivo* scans

FFC-MRI studies on human subjects have so far shown a 60% average success rate, with failures being due to claustrophobia or narrowness of the scanner bore. No scans were aborted to date due to claustrophobia once the subject was placed inside the scanner. Scan management was facilitated by the ability to switch the magnetic field off during positioning on the scanner couch and by the possibility to adjust the acquisition field according to the acquisition RF coil resonant frequency so that minimal RF coil tuning adjustments were required. During the scans the subjects usually experienced mild flashes of light in their peripheral vision, called magnetophosphenes, which are known to be due to fast varying magnetic fields and are also sometimes observed in conventional MRI^26^. Acoustic noise due to gradient switching was significantly lower than for a conventional high-field scanner and was below the noise level of quiet human speech with most pulse sequences; the actual sound level could not be measured because of the lack of an MR-safe measuring instrument. Switching of the main magnetic field resulted in no significant additional acoustic noise. Despite the lack of any significant acoustic hazard, earplugs were provided to all volunteers and patients (MR151, Wardray Premise, Surrey, UK) and were sufficient to provide a comfortable scan. The total duration of FFC-MRI scans varied between 35 and 50 min from positioning of the patient to withdrawal.

**Figure 5:**
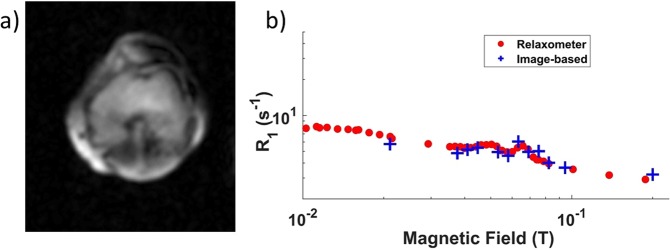
(**a**) Knee scan of a healthy volunteer with 8 mm thickness and 2 mm in-plane resolution, using a spin echo imaging sequence with 24 ms echo time. A shading artefact can be seen in the upper right corner due field inhomogeneity. (**b**) 1/T_1_ dispersion curve of the popliteus muscle measured from the image (red dots) and from a human muscle obtained from surgery (blue crosses), taken at the resection margin of a musculoskeletal sarcoma resection from the neck.

**Figure 6:**
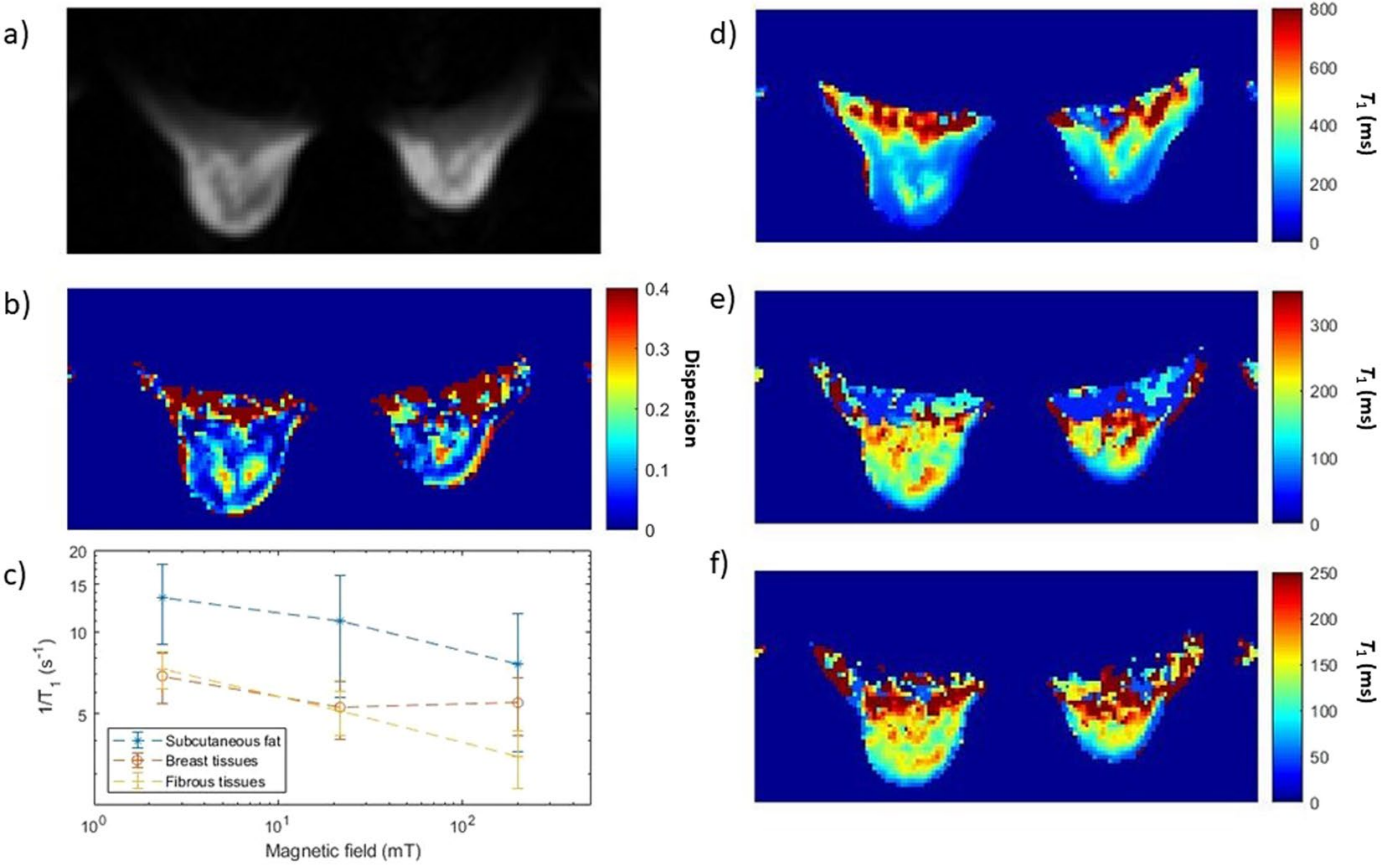
Scan of the breasts of a healthy female volunteer obtained by FCSE with FOV of 40 × 20 cm, slice thickness of 1 cm, in-plane resolution of 3 × 3 mm, echo time of 26 ms, and 1 acquisition. (**a**) Magnitude image obtained from a scan at 0.2 T; the measured signal-to-noise ratio was 113. (**b**) Dispersion image obtained from the power law exponent of the fitting model (see “Data processing” in the Methods section). (**c**) NMRD profile of the breast tissues (red), subcutaneous fat (blue) and fibrous tissues (yellow). (**d–f**) T_1_ maps at 200 mT, 20 mT and 2 mT respectively.

**Figure 7:**
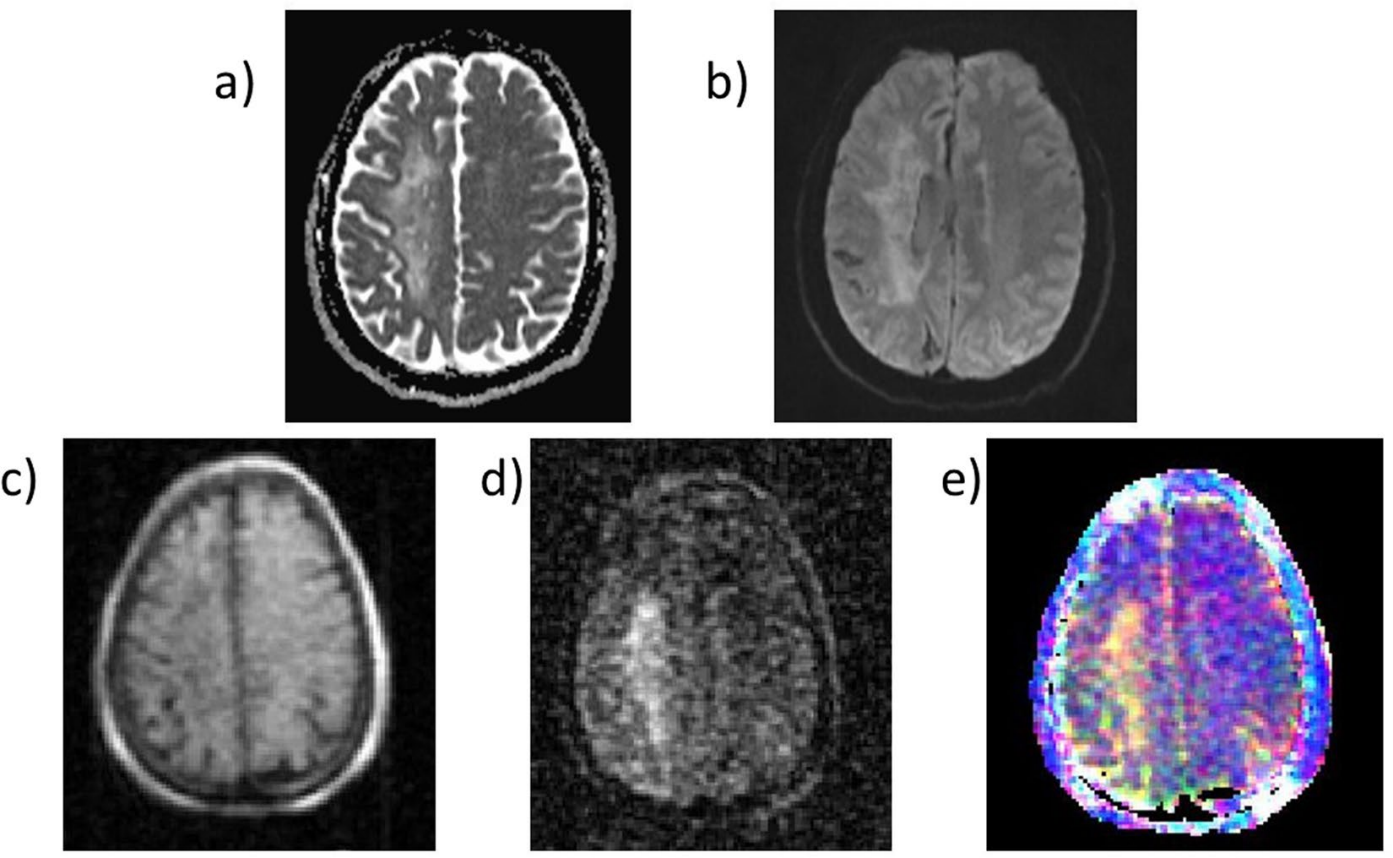
Transverse images of the brain of a patient diagnosed with vasculitis. The top images are from a 3 T MRI scanner (Achieva, Philips, Netherlands) using T_2_-weighted (**a**) and diffusion-weighted (**b**) sequences. The bottom line shows images from FFC-MRI, either magnitude images at 200 mT and 455 ms evolution field and time (**c**) or at 6.9 mT and 196 ms evolution field and time (**d**). These show large changes in the T_1_ values between these fields, with the lesion showing longer T_1_ relative to other tissues at lower fields. (**e**) shows a composite image regrouping the T_1_ images at three different fields (200, 6.9 and 1.3 mT).

A typical image of the knee of a healthy volunteer is presented in Fig. 5, along with dispersion data obtained from the image. Figure 6 shows an image of the breasts of a healthy female volunteer, together with a 1/*T*_1_-dispersion image with dispersion curves obtained from image segmentation. Finally, *T*_1_-dispersion images can be generated by mixing the *T*_1_ weighted images obtained at different fields: Fig. 7 shows an attempt by the authors to underline a lesion in the brain of a patient diagnosed with likely vasculitis, exhibiting different shades of colours (yellow, green and red) within the lesion owing to the different *T*_1_ dispersion properties within this area. This allows distinguishing different tissues according to their structures that may otherwise appear similar at fixed fields.

As mentioned previously, FFC-MRI generates *T*_1_-dispersion contrast by varying the main magnetic field so part of the imaging cycle does not meet the requirement of frequency matching between the RF coil and the Larmor frequency of the spin system. Added to the 50% duty ratio observed to remain within thermal limitation of the current amplifier, this limits the amount of data available during a scan so that image quality is generally lower than can be produced from a fixed-field scanner operating at the same maximum field strength^27^.

## Discussion

The FFC-MRI scanner presented here exhibited some relatively large field instabilities during operation, compared to fixed-field MRI. Nevertheless, clinically usable images providing reliable *T*_1_ dispersion data could still be obtained by employing post-acquisition corrections^25^. Local fluctuations of the magnetic field were also observed, reaching 0.2 μT AC and 10 μT inhomogeneous DC components due to the mains electrical supply and to ferromagnetic materials buried in the concrete floor of the scanning room, respectively. While better stability and homogeneity are desirable and are under investigation as part of our current project^28^, nevertheless the present configuration was sufficient to proceed with clinical studies and provided valuable information about the water dynamics within tissues, which will be presented in detail in future publications.

Experiments on phantoms successfully allowed differentiating between cross-linked BSA and manganese chloride solutions simply by clustering over *T*_1_ dispersion information, taking advantage of the different shapes of the dispersion curves provided by these two compounds. The average *T*_1_ value was also reliably used to determine the sample concentration (with a small bias for manganese chloride samples). This simple approach proved effective even with relatively little information from the dispersion data, through the relationship between the linearity of the dispersion amplitude and the concentration of relaxing agents, showing that FFC-MRI can not only differentiate materials easily but can also provide quantitative information about their composition. Finer approaches could be used to classify biological tissues by exploiting their significantly different dispersion profiles found *in vivo*, such as those observed between fatty tissues, fibrous tissues or subcutaneous fat in Fig. 6. Precise knowledge of the dispersion of these tissues could lead to quantitative mapping of tissue-specific *T*_1_ dispersion features in a similar manner to the method demonstrated here with phantom objects. Additionally, better magnetic-field resolution could make it possible to extract finer details of the dispersion curve, which can help separating the contributions from structurally different compounds and should provide more tissue-specific information based on their composition. This should be achieved by faster data acquisition strategies.

Scan time was also kept under reasonable limits so that human studies are possible, within the limits of the scanner size. Several studies are currently ongoing demonstrating that FFC-MRI can be used clinically and has a strong potential for medical innovation. Previous work by our group and others^2,5,18,29–36^, using biopsy tissues measured using benchtop relaxometers, showed potential biomarkers in a variety of pathologies that can now be investigated *in vivo*, using endogenous contrast. This paves the way towards quantitative molecular dynamics-based contrast for clinical biomarkers using the FFC-MRI technology to complement conventional MRI scanners. FFC-MRI also brings considerable practical advantages as it is a low magnetic field technique, which mitigates many problems arising from the use of strong magnetic fields in conventional MRI. Specifically, it substantially reduces some of the hazards associated with the “missile effect” and RF heating, and is also much less sensitive to susceptibility or chemical shift artefacts.

Finally, the data shown here represent early results; the FFC-MRI system presented in this paper can be optimised in many aspects, from the use of coil arrays to the improvement of pulse sequences or further stabilisation of the readout field. These developments are currently underway in our laboratory.

## Methods

FFC-MRI systems are similar to conventional MRI in principle and consist of: a magnet, which produces a pulsed homogeneous field where the patient lies; three gradient coils, which produce linear gradients of the magnetic field in all three directions of space; several shim coils, which are designed to compensate for small inhomogeneities in the pulsed magnetic field and to improve the image quality; an RF system, which transmits pulses of RF radiation and detects the weak NMR signals that form the image; and a console, which synchronises the system using a list of events called the pulse sequence. However, FFC-MRI differs significantly from MRI in the technologies employed and design considerations and choices are critical; these are described below.

### The magnet

The magnet is a major element of an MRI system and careful design is crucial for good performance. In FFC-MRI the magnetic field is inherently limited to fields below 0.5 T because of the typical values of *T*_1_ at these fields (several hundreds of milliseconds) coupled with the safety limitation of the rate of change of magnetic field (typically 20 T/s). The rapid variations of the magnetic field during the pulse sequence, up to 200 mT field excursion in 10 ms, prevent the use of superconducting magnets, which would quench. Resistive magnets are used instead but must present low inductance to be driven quickly, and they also waste energy by Joule heating so they require efficient cooling. In addition, they need powerful current supplies that can generate the peak power of 200 kW required to energise and de-energise the magnet during use.

In the system presented here (Fig. 1) the main magnet was made of copper windings embedded in an epoxy cylinder of 2 m length, 720 mm outer diameter and 560 mm inner diameter made as a prototype in partnership with a commercial supplier (Tesla Engineering Ltd., Sussex, UK). This cylinder also contained some elements of two of the shim coils, the rest of the shim and gradient coils residing in a second, smaller cylinder of 500 mm inner diameter, concentric with the main magnet. The main magnet winding was constructed from hollow copper conductor (to accommodate cooling fluid) with a square section of 11 mm × 11 mm and 7 mm diameter circular hole. It was laid out as a cylindrical winding of 12 mm pitch to form three independent coaxial co-wound coils. The winding of each coil was rotated around the Z-axis by 120° from each other to reduce field harmonics. Each coil was driven by 6 bipolar current amplifiers (IECO Oy, Finland, Model PA 400–350), with a total of 18 amplifiers driven simultaneously to produce up to 650 A for each of the three groups of coils. This configuration allowed reducing the total inductance of the magnet to 15.3 mH, in turn lowering the voltage required to drive the system. The dimensions of the magnet were chosen so as to reach a nominal continuous field of 0.2 T at a total current of 1950 A and drive voltage of 55.9 V (hence a total resistance of 29 mΩ) and the inductive load led to a voltage requirement of 790 V to ramp the magnet at 20 T/s, the typical safety limit imposed to avoid peripheral nerve stimulation in human subjects. In practice, the current amplifiers used could switch from 0 to 1950 A with a minimum transition time of 12 ms, corresponding to a peak power of 285 kW.

With such high currents, thermal considerations are important as thermal drifts can cause problems for image acquisition; furthermore, loss of cooling may lead to magnet failure. This magnet was designed to reach a nominal operating temperature of 60 °C (+35 °C change from ambient temperature) while generating 107 kW at 100% duty cycle. The primary cooling circuit contained an 80/20 mixture of water/ethylene glycol flowing inside the conductors at 46 L/min via 18 parallel circuits. The heat was dissipated to the atmosphere via a chiller unit (Hyfra, Krunkel, Germany, model SVK360) in a closed-loop secondary cooling circuit. The maximum temperature allowed for the magnet is 80 °C, but in practice the coolant temperature did not exceed 40 °C.

### Gradient and shim coils

The gradient coils followed typical Golay set (X, Y) and Maxwell pair (Z) designs, optimised by matrix inversion^37^ and were embedded in the previously-described inner cylinder. Each was driven by a single amplifier (AE Techron, Elkhart, IN, USA, model 7792) with maximum peak current and voltage of 107 A and 400 V and a 50 kHz bandwidth. This allowed reaching a maximum field gradient of 18, 18 and 19.4 mT/m for X, Y and Z respectively. Severe *B*_0_ field inhomogeneity required careful shimming using eleven shim coils: Z2, Z3, Z4, Z1-Z3, XY, XZ, YZ, X2-Y2, named after the spherical harmonics they correct for, in addition to the gradient coils X, Y and Z. The extra shim coil Z1-Z3 was added by the magnet manufacturer to compensate for strong Z1 and Z3 inhomogeneity components. All the shim coils were driven by separate current amplifiers (Danfisik A/S, Taastrup, Denmark, model 892 and Resonance Research Inc., Billerica, MA, USA, model MXA-8-C).

### Shielding and Earth’s field compensation

The scanner was surrounded by a Faraday cage (Rainford EMC Systems Ltd, Haydock, UK) to prevent RF interference from corrupting the images. The cage was made of copper mesh rather than copper foil, in order to reduce the potential for eddy currents during magnetic field switching, with a wire diameter of 30 μm and gap of 20 μm woven into an 80 μm-thick mesh and providing −70 dB attenuation at 8.5 MHz. All the connections into the Faraday cage were made through a penetration panel via RF filters. In addition to the Faraday cage, an RF shield was built inside the FFC-MRI scanner to avoid coupling the RF coil with the scanner coils and to reduce RF noise. This cylindrical shield was built from the same copper mesh as the Faraday cage and was split along the Z direction in order to avoid the formation of eddy currents during *B*_0_ variation. Great care was also taken to avoid using metallic parts in the vicinity of the magnet for similar reasons.

Prior to FFC-MRI measurements, static environmental magnetic fields were measured inside the scanner using four fluxgate magnetometers with a ±100 μT range (Bartington Instruments Ltd., Oxon, UK, model Mag690), one placed at the centre of the scanner and the others 15 cm away in the X, Y and Z directions, all attached to a rigid frame, connected to signal conditioning units (Bartington model PSU1) and monitored over a local network (National Instruments Corp., Austin, TX, USA, NI9220 card and cDAQ9181 unit). This allowed measuring fluctuations down to the nanotesla range, which was sufficient for our purpose. The data recorded was acquired and analysed to provide the average and first order moments of the environmental magnetic field inside the magnet. The average environmental field was minimised by manually adjusting the currents in three home-built pairs of 2-metre side square Helmholtz coils positioned along all three principal axes within the scanner, while the field gradient was adjusted using two coils, S4 and S5. S4 was constructed according to the design proposed by Lother *et al*.^38^ while S5 was equivalent to S4 with a 45° rotation along the Z-axis. Note that the Helmholtz pair aligned in the direction of *B*_0_ was split into two windings driven separately in order to avoid damaging their current supplies by the voltage spikes induced by field cycling, about 45 V in each winding.

### RF chain

RF pulse transmission and reception were performed by an 8-leg, low-pass birdcage head coil for the phantom and head imaging, a coupled pair of solenoids for breast imaging and a saddle coil for knee imaging (see Appendix for details), all incorporating 3d-printed rigid PLA support structures (Ultimaker B.V., Geldermalsen, Netherlands, model Utlimaker 3 Extended) and polycarbonate tubes. The head coil was made from 1.2 cm-wide and 127 μm-thick copper tape (Peak Dale Products Ltd., Buxton, UK) while the breast and knee coils were wound using 4 mm enamelled copper wire. Both were tuned and matched manually using variable capacitors (Sprague Goodman Electronics, Westbury, NY 11590, USA, model GME50601 (with ferromagnetic washers replaced by nylon ones) and Voltronics Inc., Chicago, Il, USA, model AP55HV). The head coil showed a loaded Q factor of 150 while that of the breast coil reached 300. The RF pulses from the console were amplified by a 2 kW amplifier (model AMT, Herley, Lancaster, UK, model 3200) that was connected to the RF coil via a home-built passive quarter-wave T/R switch; the signal received was amplified by 36 dB by a wide-band preamplifier (L3 Narda-MITEQ, Hauppauge, NY, USA, model AU1466). Before each scan, the magnetic field generated by the FFC-MRI scanner for image acquisition was adapted to the frequency of the RF antenna so that coils of any frequency up to 8.5 MHz could be used on this system, providing that the RF switch was tuned accordingly to protect the preamplifier.

### Control and synchronisation

The RF and imaging gradient systems were controlled by a commercial MRI console (MR Solutions Ltd., Guildford, UK, model EVO) to execute the pulse sequence. However, this console could not reliably control the main magnetic field and shim coils so these functions were delegated to a separate computer (National Instruments Corp., Austin, TX, USA, model cDAQ-9139, referred to hereafter as the “cDAQ computer”). The main magnetic field was controlled by a ±10 V analogue signal generated by a custom-made 16-bit DAC (Oxford Instruments plc, Abingdon, UK) with a 3 μT equivalent field resolution via a digital control card (National Instruments, model NI9403), while the shim coils were controlled directly from a dedicated card (National Instruments, model NI9139). Synchronisation between the computer and console was done using digital connections with TTL logic, which was fast enough to neglect propagation and processing delays.

The two systems were connected to the operator computer that programmed each one separately, started them synchronously and collected data on-the-fly using MATLAB 2017a (Mathworks Inc., Natick, MA, USA), using ActiveX controls to operate the console and TCP/IP to control the cDAQ computer. Prior to starting a pulse sequence the associated variation of the main field (the field-cycling) were processed into a waveform that was sent to the cDAQ computer so that field profiles were easy to design and could be changed arbitrarily using MATLAB code. Each pulse sequence was assigned a MATLAB file that contained all the code needed to generate the corresponding field waveform, store the data during the acquisition and process that data to form the image. This system also allowed controlling the shim currents during the field ramps so that the magnetic field remained homogeneous inside the imaging volume during field-cycling, even at very low fields. This was done by scaling the current in each shim coil according to the main field waveform’s value, using correction factors found at minimum and maximum fields from preliminary measurements. A Graphical User Interface (GUI) was programmed in MATLAB to facilitate the routine use of the scanner.

### Pulse sequences

The system’s console included a range of conventional MRI pulse sequences, some of which have been adapted by us to include the magnetic field waveforms necessary for FFC-MRI. Our primary imaging sequence is an adapted version of the commonly-used spin-echo sequence which offers a reliable method of assessing the system’s performance. Other sequences, including rapid imaging methods, are compatible with FFC-MRI, provided the SNR penalties associated with such techniques do not exceed tolerable levels. We and other groups have previously presented work describing the implementation of rapid imaging sequences on similar low field or field-cycling MRI scanners^39–42^.

Both the main magnet and its power supplies were sensitive to thermal drifts. To correct these, a “navigator” free induction decay (FID) acquisition was integrated into the imaging pulse sequences and measured on-the-fly during image acquisition. The Fourier transform of this signal provided an accurate estimation of the magnetic field experienced by the spin system which was sent immediately to the cDAQ computer to correct the drifts for the next acquisition. This allowed correction of the slow thermal drift of magnetic field observed using the system within the resolution of the 16-bit DAC that controlled the main magnetic field (3 μT, or equivalently ±130 Hz).

A shimming pulse sequence was also developed to correct for inhomogeneities of the main magnet (*B*_0_). These inhomogeneities proved not to be easily amenable to standard correction techniques (such as FASTERMAP^43^) therefore we used an iterative method relying on the maximisation of the FID amplitude after a saturation pulse. This was implemented using a teacher-learner algorithm^44^ with 6 learners and a maximum of 50 iterations.

### Materials and subjects

A “bottle phantom” was designed using seven bottles of 35 mm diameter and 60 mm length. Four bottles were filled with solutions of increasing concentration of manganese chloride (0.38, 0.52, 0.74 and 1.00 mM) to provide a range of *T*_1_ values and dispersions, while the other three contained a mixture of 8% formaldehyde with bovine serum albumin at 5%, 10% and 20% w/w concentrations to generate cross-linked protein gels that exhibited quadrupolar peaks in their dispersion curves as shown in the literature^45^. Reference measurements of the *T*_1_ dispersion curves were made at room temperature using a commercial field-cycling relaxometer (Stelar S.r.l., Mede, Italy, model SMARTracer) and compared with the spin-echo method presented earlier.

All patients and volunteers provided informed consent to be imaged by our FFC-MRI scanner and for the anonymous use of their data and their publication. In particular, informed consent was obtained to publish the identifiable image presented on Fig. 1 in an online open-source journal. All our clinical studies have been approved either by the North of Scotland Research Ethics Committee (NoSREC) or the local College Ethics Review Board (CERB) ethics committee and all the methods described were performed in accordance with the Good Clinical Practice guidelines and regulations defined by Medicines and Healthcare products Regulatory Agency (MHRA), UK. These studies were still ongoing at the time of writing. Patient access and management are important aspects of the design of an MRI scanner and full MRI safety screening was conducted on each participant in preparation for the scan. The small magnet bore limited access to non-claustrophobic, fit persons but was mitigated by adding in-bore lighting and air flow inside the scanner. The couch was custom-made (PPS Glass Fibre Ltd., Inverurie, UK) out of fibreglass and slid manually on PTFE rails; it was tested for safety according to International Electrotechnical Commission (IEC) medical devices directive IEC 60601-2-33. Patient access to the couch was provided by MRI-safe steps (Wardray Premise, Thames Ditton, UK, model MR2505) and communication with the scanner operator was achieved via a condenser microphone (Kingstate Electronics Corp., New Taipei City, Taiwan, model KEIG4537TFL-N) and pneumatic headphones (Wardray Premise, Thames Ditton, UK, model MR151). A panic alarm was also fitted for emergencies (Sensorium Ltd., Dunfermline, UK). An MR-safe health monitoring system (Bayer plc, Leverkusen, Germany, model Medrad Versis) was also installed inside the shielded enclosure for patient monitoring during scans.

### Data processing

Magnetic field fluctuations during signal acquisition were measured using the phase of the FID signal, after linear correction to account for the field offset. The error was defined as being three times the standard deviation of the corrected phase data measured over the first 10 ms of the signal, which provided an estimation of the field stability in ppm.

Variations in the main magnetic field during the acquisition stage were responsible for artefacts in the phase-encode direction because of the lack of reproducibility of the phase value from one acquisition of k-space to the next, introducing a random phase term in the data in each k-space line. A correction technique was developed that could correct for this effect, provided that the image contained some empty space, as already published by our group^25^. The control software was coded in MATLAB 2017a and integrated into the data processing pipeline automatically at the end of each acquisition using parallel processing to allow the user to continue experimentation while image processing continued in the background.

Estimation of phantom material content was performed using segmentation of the FFC-MRI images using MATLAB 2017a. Clustered data were regrouped and analysed to provide their average *T*_1_ dispersion curve, which was then compared to reference data comprising high-quality NMRD profiles of MnCl_2_ solutions and cross-linked BSA samples of known concentration previously obtained by FFC-NMR. The best model was selected to minimise the sum of the residuals squared as the decision criterion after adjusting the mean dispersion, which was used to determine the concentration once the model was selected.

SNR was measured from images using the ratio between the average magnitude signal over the standard deviation of the background signal, multiplied by 0.655 to account for the Rician distribution of noise in magnitude MRI images^46^.

The dispersion image presented in Fig. 6b was prepared from the *T*_1_ dispersion in the breast tissues, evaluated from the magnitude images obtained at different magnetic fields. *T*_1_ dispersion data was analysed by curve fitting using a power law:

$$\frac{1}{{T}_{1}}=a\,{{f}_{L}}^{b}$$

where *f*_L_ is the Larmor frequency. This model was observed to fit such tissues from previous studies on resections done by our group, and the exponent *b* was plotted to provide the map shown in the manuscript.

## Supplementary information

Supplementary information
