# Supplementary material for: A whole-body Fast Field-Cycling scanner for clinical molecular imaging studies
Source: Sci Rep. 2019 Jul 18;9:10402. doi: 10.1038/s41598-019-46648-0 (PMC6639535; doi:10.1038/s41598-019-46648-0)
Supplement: Supplementary file 1 — Supplementary information [file 41598_2019_46648_MOESM1_ESM.docx]

A whole-body Fast Field-Cycling scanner for clinical molecular imaging studies

# Author list and affiliations

Lionel M. Broche^*1^, P. James Ross^1^, Gareth R. Davies^1^, Mary-Joan MacLeod ^2^, David J. Lurie^1^

^1^Aberdeen Biomedical Imaging Centre, Biomedical Physics Building, Foresterhill, AB25 2ZD, Aberdeen, U.K

^2^Acute Stroke Unit, Aberdeen Royal Infirmary, Foresterhill, AB25 2ZD, Aberdeen, U.K

# Corresponding author details

Email: l.broche@abdn.ac.uk

Tel: 01224 437833

Address:

Biomedical Physics Building

University of Aberdeen

Foresterhill

Aberdeen

AB25 2ZD

United Kingdom

# Additional Information

## Appendix: dimensional and functional specifications

### Main magnet specifications

| Conductor width (mm) | 11 |
| --- | --- |
| Conductor Height (mm) | 11 |
| Hole Diameter (mm) | 7 |
| Length (m) | 1095 |
| RMS Current (A) | 650 |
| Water inlet temperature (deg C) | 25 |
| Target outlet temperature (degC) | 60 |
| Pressure (bar) | 2 |
| Circuits | 18 |
| Resistivity (nohm.metres) @ 20C | 17.2 |
| T_coeff_ (nohm.metres/degC) | 0.083 |
| Assumed mean resistivity (nohm.metres) | 19.1 |
| Cross-section (mm^2^) | 82.5 |
| Resistance (ohms) | 0.083 |
| Inductance (mH) | 15 |
| Water heat capacity (J/K/litre) | 4200 |
| Mass of copper | 804 |
| Flow (litres/min) | 47 |
| Dissipation (W) | 107129 |
| DC tension (V) | 165 |
| Nominal temperature difference (K) | 32.9 |
| Maximum temperature difference (K) | 57.9 |

### Gradient coils

"DSV" stands for "diameter spherical volume". Spatial distortion is evaluated at an array of points on the surface of such a sphere. For an X gradient of central strength G_X_^c^ at a given current, the percentage spatial distortion at evaluation point (x_i_, y_i_, z_i_) is defined as:

$$\% error = 100\times\frac{\left( B_{z}\left( x_{i},y_{i},z_{i} \right)-G_{X}^{C}\times x_{i} \right)}{G_{X}^{C}\times DSV/2}$$

The rise-time *Δt* is quoted from measurements of the 98% response time.

|  | X and Y | Z |
| --- | --- | --- |
| Strength per amp (μT/m/A) | 90.0 ±5% | 97 ±5% |
| Spatial distortion  over 35cm DSV | ±3% | -3.4% +2.3% |
| X-Y orthogonality | 90º±0.1º | N/A |
| Inductance (μH) | 165 ±5% | 180 ±5% |
| DC resistance (mΩ) | <50 | <45 |
| Peak strength (mT/m) | 31.5 ±5% | 33.9 ±5% |
| Rise-time (0-98%) (μs) | 175 ±5% | 190 ±5% |
| Slew Rate (T/m/s) | 180 | 178 |
| Peak operating voltage (balanced drive) (V) | 400 | 400 |
| Peak current (A) | 350 | 350 |
| Max. temperature (ºC) | 70 | 70 |

### Shim coils

| Axial shims | Z^2^,Z^3^,Z^4^ |
| --- | --- |
| Transverse shims | ZX, ZY, X^2^-Y^2^, XY |
| Shim operating current | 10 A per channel |
| Shim supply type | Wide bandwidth current source, 100 V compliance |

| Shim | Strength on 35cm DSV (μT/A) | Peak current (A) | Resist­ance (Ω) | Induct­ance (mH) | Purity on 35cm DSV | Mutual coupling |
| --- | --- | --- | --- | --- | --- | --- |
| Z^2^ | 26 | 10 | 1.5 | 4.6 | 3% | 60 μH to magnet |
| Z^3^ | 8.9 | 10 | 0.7 | 2.4 | 6% | 10 μH to gradient |
| Z^4^ | 6.5 | 10 | 1.8 | 5.1 | 9% | 20 μH to magnet |
| ZX | 11 | 10 | 1 | 2.6 | 1.5% | N/A |
| ZY | 11 | 10 | 1 | 2.6 | 1.5% | N/A |
| X^2^-Y^2^ | 9.5 | 10 | 2 | 4.2 | 1.5% | N/A |
| XY | 9.5 | 10 | 2 | 4.2 | 1.5% | N/A |

### Radiofrequency antenna

|  | Head coil | Breast coil | Knee coil |
| --- | --- | --- | --- |
| Design | Low-pass birdcage, 8 rungs | Adjacent solenoids, 6 turns each, connected in series | Saddle coil |
| Frequency (MHz) | 8.3 | 8.0 | 8.3 |
| Q factor (unloaded/loaded) | 210/110 | 300/150 | 320/160 |
| Ringing time (μs) | 11 | 12 | 13 |
| Dimensions (cm) | Diameter: 23  Length: 25 | i.d. 14  8 | i.d. 17  22 |
| Conductor | Copper sheet 200μm thick, 2cm width |  |  |
